# Opioid receptor agonists may favorably affect bone mechanical properties in rats with estrogen deficiency-induced osteoporosis

**DOI:** 10.1007/s00210-016-1295-6

**Published:** 2016-11-28

**Authors:** Aleksandra Janas, Joanna Folwarczna

**Affiliations:** Department of Pharmacology, School of Pharmacy with the Division of Laboratory Medicine in Sosnowiec, Medical University of Silesia, Katowice, Poland

**Keywords:** Opioids, Osteoporosis, Bone mechanical properties, Rats

## Abstract

The results of epidemiological, clinical, and in vivo and in vitro experimental studies on the effect of opioid analgesics on bone are inconsistent. The aim of the present study was to investigate the effect of morphine (an agonist of opioid receptors), buprenorphine (a partial μ opioid receptor agonist and κ opioid receptor antagonist), and naloxone (an antagonist of opioid receptors) on the skeletal system of female rats in vivo*.* The experiments were carried out on 3-month-old Wistar rats, divided into two groups: nonovariectomized (intact; NOVX) rats and ovariectomized (OVX) rats. The bilateral ovariectomy was performed 7 days before the start of drug administration. Morphine hydrochloride (20 mg/kg/day *s.c.*), buprenorphine (0.05 mg/kg/day *s.c.*), or naloxone hydrochloride dihydrate (2 mg/kg/day *s.c.*) were administered for 4 weeks to NOVX and OVX rats. In OVX rats, the use of morphine and buprenorphine counteracted the development of osteoporotic changes in the skeletal system induced by estrogen deficiency. Morphine and buprenorphine beneficially affected also the skeletal system of NOVX rats, but the effects were much weaker than those in OVX rats. Naloxone generally did not affect the rat skeletal system. The results confirmed the role of opioid receptors in the regulation of bone remodeling processes and demonstrated, in experimental conditions, that the use of opioid analgesics at moderate doses may exert beneficial effects on the skeletal system, especially in estrogen deficiency.

## Introduction

There is a progressive increase in the long-term use of opioid analgesics in the treatment of pain associated with cancer and other diseases, such as degenerative changes in the musculoskeletal system (Cherubino et al. 2012). More and more patients, including the elderly, in whom bone mass physiologically decreases, undergo prolonged exposure to opioid analgesics (agonists, partial agonists and agonist-antagonists of opioid receptors) (Ballantyne 2012). This applies particularly to postmenopausal women who are at increased risk of developing osteoporosis due to estrogen deficiency (Braden et al. 2012).

Endogenous opioid peptides and their receptors (μ, δ, and κ) are present in the skeletal system (Baldock et al. 2012; Böhm and Grässel 2012; Spetea 2013), but the role of endogenous opioid peptides in the regulation of bone remodeling processes and the effect of opioid analgesics on the skeletal system have not been fully clarified yet.

Administration of opioid analgesics may adversely affect the skeletal system; however, the results of epidemiological and clinical studies, as well as in vivo and in vitro experimental studies published so far are inconsistent. The population-based and clinical studies indicate rather damaging effects of opioids on bone leading to a reduction in bone mineral density (BMD) (Pedrazzoni et al. 1993; Kim et al. 2006; Fortin et al. 2008; Dürsteler-MacFarland et al. 2011; Grey et al. 2011; Duarte et al. 2013) and increased risk of fracture (Guo et al. 1998; Ensrud et al. 2003; Vestergaard et al. 2006; Saunders et al. 2010; Solomon et al. 2010; Miller et al. 2011; Carbone et al. 2013; Li et al. 2013), although recently two reports indicating possible favorable opioid effects on the skeletal system in women have been published (Vestergaard et al. 2012; Lee et al. 2013). The unfavorable effects of opioids on the skeletal system are usually attributed to inhibitory effects on the endocrine system (hypogonadism), as well as increased tendency to falls (Vestergaard et al. 2006; Daniell 2008; Saunders et al. 2010; Brennan 2013; Duarte et al. 2013); however, results of a small number of in vitro and in vivo experimental studies suggest that opioids may also act directly on bone tissue and exert differential effects (Hall et al. 1996; Pérez-Castrillón et al. 2000; King et al. 2007; Akhoundi et al. 2010; Bastos et al. 2011; Boshra 2011; Ezzatabadipour et al. 2011; Chrastil et al. 2013).

The aim of the present study was to verify, in experimental conditions, the widely held view that opioid analgesics unfavorably affect the skeletal system. The skeletal effects of morphine (an agonist of opioid receptors) and buprenorphine (a partial μ opioid receptor agonist and a κ opioid receptor antagonist), widely used in the pharmacotherapy of pain, were examined in mature female rats. Moreover, the effects of naloxone (an antagonist of opioid receptors) were studied. Since, due to cessation of ovarian estrogen production in women at menopause, the opioids act on the female organism in different estrogen environment, the present study was conducted in two experimental models: nonovariectomized (with normal levels of estrogen) rats and bilaterally ovariectomized (estrogen-deficient) rats.

## Materials and methods

### Animals and chemicals

The experiments were carried out on 3-month-old female Wistar rats obtained from the Center of Experimental Medicine, Medical University of Silesia, Katowice. The rats were fed a standard laboratory diet Labofeed B (Wytwórnia Pasz “Morawski”, Poland). The protocol for the experiments on animals was approved by Local Ethics Commission, Katowice, Poland.

Drugs used were as follows: morphine hydrochloride (substance, Kutnowskie Z.F. “Polfa” S.A.) at a dose 20 mg/kg *s.c.* daily; buprenorphine hydrochloride (Bunondol, ampoules, Polfa Warszawa) at a dose 0.05 mg of buprenorphine/kg *s.c.* daily; naloxone hydrochloride dihydrate (substance, Sigma-Aldrich) at a dose 2 mg/kg *s.c.* daily; ketamine (Bioketan, Vetoquinol Biowet); and xylazine (Rometar, Spofa). Morphine and buprenorphine were administered at effective analgesic doses for rats (Mucha et al. 1996; Allen and Dykstra 2000; Curtin et al. 2009). Naloxone was administered at a dose used previously in rats to block the opioid receptors (Farhadinasab et al. 2009).

**Fig. 1:**
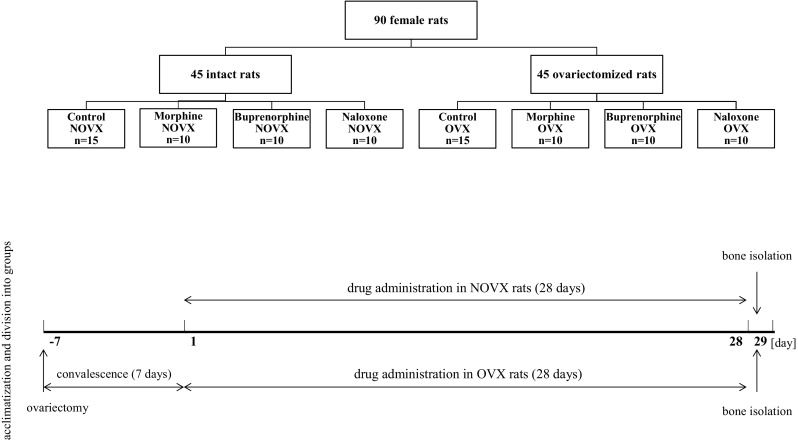
Group assignments and timeline of the experiments. Morphine hydrochloride (20 mg/kg *s.c.*), buprenorphine hydrochloride (0.05 mg of buprenorphine/kg *s.c.*), and naloxone hydrochloride dihydrate (2 mg/kg *s.c.*) were administered once daily for 4 weeks

The studies were carried out on nonovariectomized (NOVX) rats and ovariectomized (OVX) rats (Fig. 1). The rats were acclimated for 7 days after delivery and then divided into experimental groups. The bilateral ovariectomy was performed 7 days before the start of drug administration, under ketamine-xylazine (*i.p.*) anesthesia. Animals were divided into eight groups: I NOVX control rats (*n* = 15), II NOVX rats treated with morphine (*n* = 10), III NOVX rats treated with buprenorphine (*n* = 10), IV NOVX rats treated with naloxone (*n* = 10), V OVX control rats (*n* = 15), VI OVX rats treated with morphine (*n* = 10), VII OVX rats treated with buprenorphine (*n* = 10), and VIII OVX rats treated with naloxone (*n* = 10). The rats from control groups were given the vehicle (0.9 % NaCl solution). The drugs or 0.9 % NaCl were administered to the rats subcutaneously, once daily for 4 weeks, at a volume of 1 ml/kg.

After 4 weeks of daily administration of the examined drugs, the animals, after overnight fasting, were anesthetized (ketamine-xylazine) and sacrificed by cardiac exsanguination. Blood samples were collected, and after clotting, serum was centrifuged (microcentrifuge type 320) and divided into samples. Samples were stored at −80 °C. The tibial and femoral bones, and L-4 vertebras, as well as the uterus and thymus were dissected free, and cleaned of soft tissue. The left tibias and femurs were immediately weighed (analytical scale AS 200 S, Ohaus, the accuracy of the measurements 0.1 mg), and their length and the diameter at the midlength were measured (digital caliper VOREL 15240, Toya, the accuracy of the measurements 0.01 mm). The vertebra, uterus, and thymus were weighed.

The left tibia, left femur, L-4 vertebra, and the proximal part of the right femur were wrapped in gauze soaked in 0.9 % NaCl solution and stored below −18 °C for further studies (Turner and Burr 1993).

### Bone mechanical properties studies

The measurements of bone mechanical properties were performed using Instron 3342 apparatus (measuring range 0–500 N). To evaluate the mechanical strength of the left femoral diaphysis and left tibial metaphysis, three-point bending tests were performed. A compression test was used to determine the strength of the right femoral neck. The data obtained during the measurements were analyzed using the Bluehill 2 version 2.14 software. The frequency of sampling was 100 Hz.

**Fig. 2:**
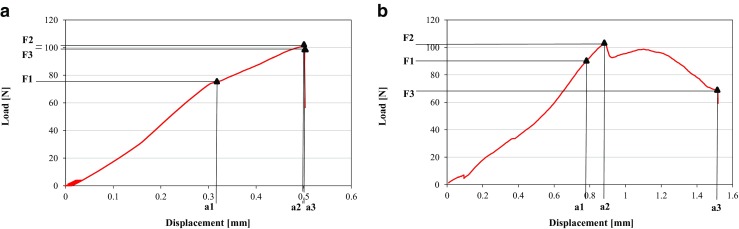
Load-displacement curves from bone bending tests performed with the use of Instron 3342 apparatus (Bluehill 2 version 2.14 software); **a** femoral diaphysis, **b** tibial metaphysis. The load, displacement, energy absorbed, and stress were determined at 3 points: the yield point (0.05 % offset), the maximum load point, and the fracture point (details in the text). *F1*, *a1*—load and displacement at the yield point; *F2*, *a2*—load and displacement at the maximum load point; *F3*, *a3*—load and displacement at the fracture point

In the bending tests, values of extrinsic parameters, depending on the dimensions of the bones (load, displacement and the energy, which was absorbed in the range of load from 0 to the given load point) were determined at 3 points: the yield point (0.05 % offset), the maximum load point, and the fracture point (Fig. 2). The intrinsic parameters, independent of the size of the bones (Young’s modulus and stress), were also evaluated.

In order to determine the strength of the left femoral diaphysis, the bone was placed on supporting points (distance 20 mm), and the load was directed perpendicularly to the long axis of the femur in the midlength of the bone (Turner and Burr 1993). To obtain steady positioning, the bone was preconditioned (five cycles of the load 0–4 N) (Westbroek et al. 2007; Folwarczna et al. 2013). The displacement rate was 0.01 mm/s. In order to determine the Young’s modulus and the stress values, it was assumed that the femoral diaphysis was an elliptical pipe (Kiebzak et al. 1988). To determine the moment of inertia, necessary for calculations, the transverse cross sections of the right femoral diaphysis were made in the midlength. The internal and external diameters of the diaphysis were measured using Osteomeasure XP v1.3.0.1 software.

The measurement of the strength of the proximal metaphysis of the left tibia was performed by applying the load directed perpendicularly to the long axis of the bone, 3 mm from the edge of the bone deprived of the proximal epiphysis (Stürmer et al. 2006; Folwarczna et al. 2013). The bone was stabilized by pre-load of 1 N. The displacement rate was 0.01 mm/s. In order to determine the Young’s modulus and the stress values, it was assumed that the cross section of the bone at the fracture site had the shape of a circle with a diameter calculated as the mean value of the diameter measured in the frontal and sagittal planes.

To determine the femoral neck strength, the diaphysis, cut at the midlength of the femur, was fixed in a methacrylate plate, and the load was applied to the head of femur (after preload of 1 N), with the displacement rate of 0.01 mm/s (Pytlik et al. 2004; Folwarczna et al. 2013). The maximum load was measured.

### Bone mineralization studies

The left tibias, left femurs, and L-4 vertebras were ashed at 640 °C for 48 h in a muffle furnace and weighed to determine bone mineral mass. The ratio of bone mineral mass to bone mass was calculated. Calcium and phosphorus content in the bone mineral were determined spectrophotometrically, using kits produced by Pointe Scientific, Inc. (Folwarczna et al. 2013). Before measurements, the mineralized bones were dissolved in 6 M HCl and then diluted in distilled water.

### Biochemical studies

Serum concentrations of biochemical markers of bone formation (osteocalcin) and bone resorption (C-terminal telopeptides of type I collagen (CTX-I)) were measured by enzyme immunoassays (Rat-MID Osteocalcin EIA and RatLaps EIA, respectively, produced by Immunodiagnostic Systems Ltd). Serum tartrate-resistant acid phosphatase 5b (TRACP 5b; RatTRAP Assay, Immunodiagnostic Systems Ltd), serum estradiol (Mouse/Rat’s Estradiol ELISA, Calbiotech Inc.), and serum calcitonin gene-related peptide (CGRP; Enzyme-linked Immunosorbent Assay Kit For Calcitonin Gene Related Peptide, USCN Life Science Inc.) levels were also determined. Moreover, serum calcium concentration was measured spectrophotometrically, using a kit produced by Pointe Scientific, Inc.

### Statistical analysis

The results are presented as means ± SD. Kruskal-Wallis ANOVA followed by Mann-Whitney *U* test were used for statistical evaluation of the results. To evaluate the effects of estrogen deficiency in control rats, the results obtained in the OVX control rats were compared with those of the NOVX control rats. The results obtained in NOVX rats treated with morphine, buprenorphine, or naloxone were compared with those of the NOVX control rats, and the results obtained in OVX rats treated with morphine, buprenorphine, or naloxone were compared with those of the OVX control rats.

## Results

### Effects of estrogen deficiency on the skeletal system

Bilateral ovariectomy caused a profound decrease in the uterus mass/body mass ratio and increases in the thymus mass/body mass ratio and body mass gain compared to the NOVX control rats (Table 1).

**Table 1:** Effects of morphine hydrochloride (20 mg/kg *s.c.*), buprenorphine hydrochloride (0.05 mg of buprenorphine/kg *s.c.*), and naloxone hydrochloride dihydrate (2 mg/kg *s.c.*), administered for 4 weeks, on the body mass gain, mass of estrogen-dependent organs, and serum levels of estradiol, calcium, and CGRP in nonovariectomized and ovariectomized rats

Parameter/group	Nonovariectomized (NOVX) rats				Ovariectomized (OVX) rats			
	Control	Morphine	Buprenorphine	Naloxone	Control	Morphine	Buprenorphine	Naloxone
Initial body mass of rats (g)	232.4 ± 11.8	230.8 ± 11.8	233.3 ± 12.6	232.6 ± 15.9	234.4 ± 12.7	233.8 ± 11.8	233.1 ± 14.1	234.8 ± 17.1
Body mass gain after 4 weeks (g)	18.7 ± 4.9	12.0 ± 3.6**	11.3 ± 9.9*	21.8 ± 8.4	51.4 ± 7.2^^^	11.9 ± 12.5^###^	26.9 ± 9.7^###^	49.2 ± 9.0
Uterus mass (g/100 g body mass)	0.144 ± 0.051	0.128 ± 0.027	0.146 ± 0.062	0.161 ± 0.057	0.028 ± 0.004^^^	0.028 ± 0.003	0.030 ± 0.004	0.028 ± 0.004
Thymus mass (g/100 g body mass)	0.135 ± 0.017	0.110 ± 0.013***	0.136 ± 0.024	0.134 ± 0.025	0.243 ± 0.025^^^	0.172 ± 0.037^###^	0.222 ± 0.037	0.235 ± 0.032
Estradiol (pg/mL)	9.86 ± 10.16	14.57 ± 7.45	14.35 ± 10.68	15.66 ± 12.02	6.00 ± 1.15	6.65 ± 1.63	7.26 ± 5.59	7.39 ± 3.12
CGRP (pg/mL)	10.86 ± 1.53	11.48 ± 0.59	11.24 ± 0.50	12.18 ± 3.52	12.87 ± 6.19	12.45 ± 1.76	11.58 ± 1.55	12.13 ± 2.18
Calcium (mg/100 mL)	10.73 ± 0.71	10.56 ± 0.64	11.30 ± 0.52	11.31 ± 0.65	11.09 ± 0.38	10.72 ± 0.60	10.50 ± 0.81	10.62 ± 0.76

Results are presented as means ± SD

*CGRP* calcitonin gene-related peptide

*p < 0.05, **p < 0.01, ***p < 0.001, significantly different from the NOVX control rats; ^^^*p* < 0.001, significant difference between the NOVX and OVX controls; ^###^ *p* < 0.001, significantly different from the OVX control rats

**Fig. 3:**
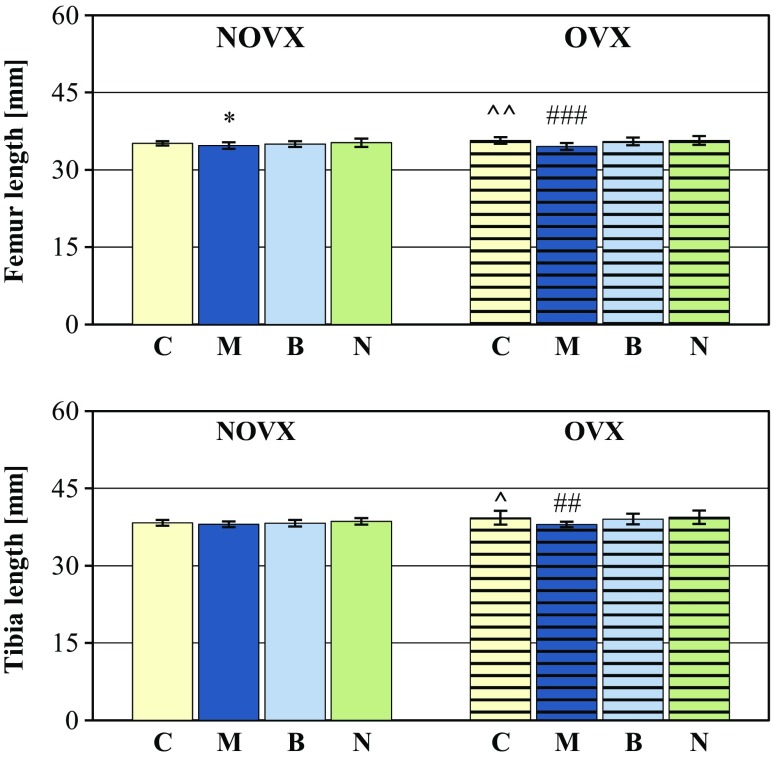
Effects of morphine hydrochloride (*M*; 20 mg/kg *s.c.*), buprenorphine hydrochloride (*B*; 0.05 mg of buprenorphine/kg *s.c.*), and naloxone hydrochloride dihydrate (*N*; 2 mg/kg *s.c.*), administered for 4 weeks, on long bone length in nonovariectomized (NOVX) and ovariectomized (OVX) rats. Results are presented as means ± SD. ^*p* < 0.05, ^^*p* < 0.01, significant differences between the NOVX and OVX controls (*C*); *p < 0.05, significantly different from the NOVX control rats; ^##^ *p* < 0.01, ^###^ *p* < 0.001, significantly different from the OVX control rats

The longitudinal bone growth was intensified in the estrogen-deficient rats (increases in the length of the tibia and femur; Fig. 3). There was no effect of estrogen deficiency on the bone mass and mass of bone mineral (not shown); however, bone mineralization was worsened (there was a significant decrease in the bone mineral mass/bone mass ratio in the L-4 vertebra and a tendency to decrease the ratio in other bones). The content of calcium and phosphorus in the bone mineral was not altered (Table 2).

**Table 2:** Effects of morphine hydrochloride (20 mg/kg *s.c.*), buprenorphine hydrochloride (0.05 mg of buprenorphine/kg *s.c.*), and naloxone hydrochloride dihydrate (2 mg/kg *s.c.*), administered for 4 weeks, on bone mineralization in nonovariectomized and ovariectomized rats

Parameter/group		Nonovariectomized (NOVX) rats				Ovariectomized (OVX) rats			
		Control	Morphine	Buprenorphine	Naloxone	Control	Morphine	Buprenorphine	Naloxone
Bone mineral mass/bone mass ratio	Femur	0.440 ± 0.016	0.453 ± 0.014*	0.457 ± 0.013*	0.459 ± 0.012**	0.435 ± 0.011	0.442 ± 0.011	0.442 ± 0.009	0.435 ± 0.011
	Tibia	0.443 ± 0.012	0.451 ± 0.006	0.451 ± 0.011	0.442 ± 0.013	0.436 ± 0.013	0.445 ± 0.009^#^	0.443 ± 0.008^#^	0.432 ± 0.007
	L-4 vertebra	0.423 ± 0.017	0.429 ± 0.024	0.431 ± 0.012	0.435 ± 0.010	0.409 ± 0.011^	0.407 ± 0.035	0.417 ± 0.022^#^	0.409 ± 0.027
Calcium content (g/g of bone mineral)	Femur	0.361 ± 0.011	0.360 ± 0.010	0.361 ± 0.013	0.366 ± 0.012	0.360 ± 0.034	0.362 ± 0.013	0.363 ± 0.010	0.367 ± 0.011
	Tibia	0.364 ± 0.011	0.373 ± 0.014	0.369 ± 0.009	0.371 ± 0.007	0.366 ± 0.007	0.370 ± 0.007	0.362 ± 0.007	0.363 ± 0.013
	L-4 vertebra	0.360 ± 0.010	0.369 ± 0.006	0.365 ± 0.006	0.362 ± 0.011	0.361 ± 0.013	0.366 ± 0.005	0.367 ± 0.007	0.362 ± 0.010
Phosphorus content (g/g of bone mineral)	Femur	0.161 ± 0.006	0.159 ± 0.005	0.160 ± 0.003	0.161 ± 0.005	0.165 ± 0.009	0.162 ± 0.006	0.162 ± 0.003	0.163 ± 0.005
	Tibia	0.161 ± 0.006	0.160 ± 0.003	0.160 ± 0.003	0.162 ± 0.003	0.163 ± 0.003	0.161 ± 0.001	0.160 ± 0.004	0.161 ± 0.003
	L-4 vertebra	0.159 ± 0.005	0.161 ± 0.003	0.160 ± 0.004	0.162 ± 0.003	0.158 ± 0.006	0.161 ± 0.002	0.161 ± 0.003	0.160 ± 0.007

Results are presented as means ± SD

*p < 0.05, **p < 0.01, significantly different from the NOVX control rats; ^*p* < 0.05, significant difference between the NOVX and OVX controls; ^#^ *p* < 0.05, significantly different from the OVX control rats

**Fig. 4:**
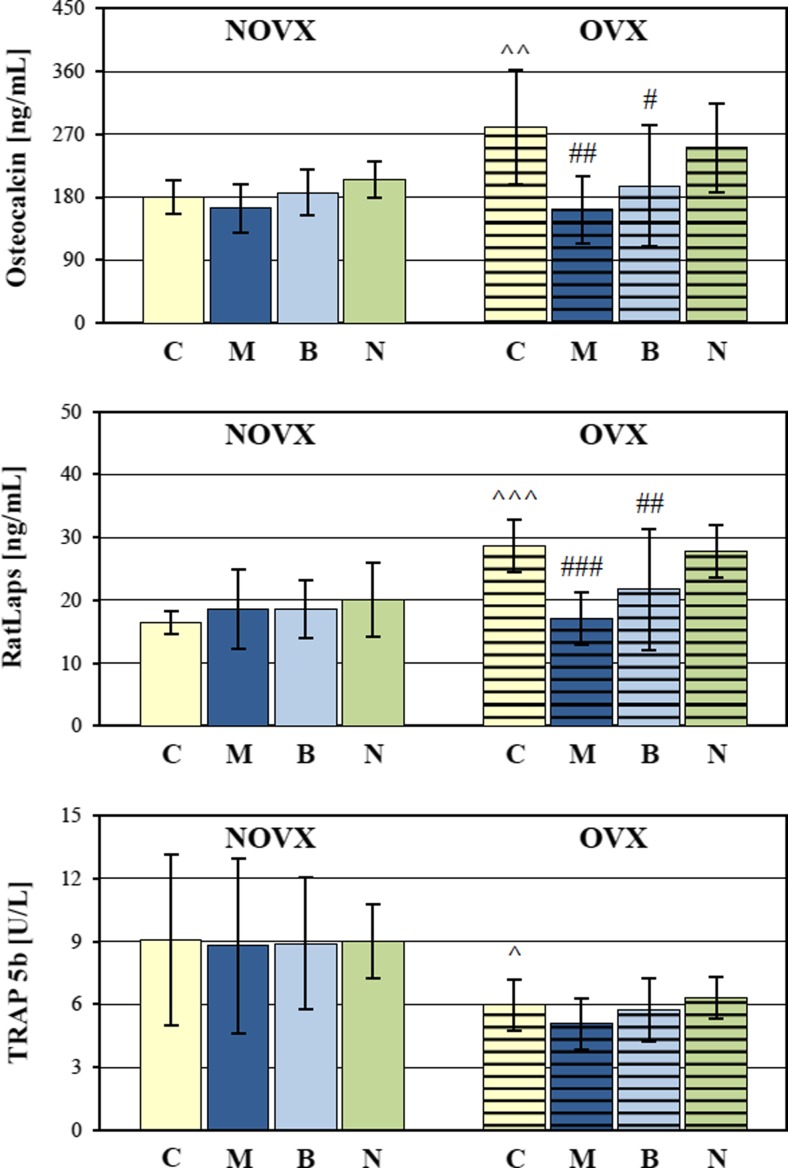
Effects of morphine hydrochloride (*M*; 20 mg/kg *s.c.*), buprenorphine hydrochloride (*B*; 0.05 mg of buprenorphine/kg *s.c.*), and naloxone hydrochloride dihydrate (*N*; 2 mg/kg *s.c.*), administered for 4 weeks, on serum levels of bone turnover markers in nonovariectomized (NOVX) and ovariectomized (OVX) rats. Results are presented as means ± SD. *RatLaps* C-terminal telopeptides of type I collagen released during bone resorption, *TRAP* tartrate-resistant acid phosphatase. ^*p* < 0.05, ^^*p* < 0.01, ^^^*p* < 0.001, significant differences between the NOVX and OVX controls (*C*); ^#^ *p* < 0.05, ^##^ *p* < 0.01, ^###^ *p* < 0.001, significantly different from the OVX control rats

Bone turnover was significantly increased in the OVX control rats, as demonstrated by increases in the serum concentrations of osteocalcin (bone formation marker) and CTX-I (bone resorption marker). A decrease in the activity of TRAP 5b, indicating a reduction in absolute osteoclast number due the decreased bone mass (Rissanen et al. 2008) was also observed (Fig. 4).

**Fig. 5:**
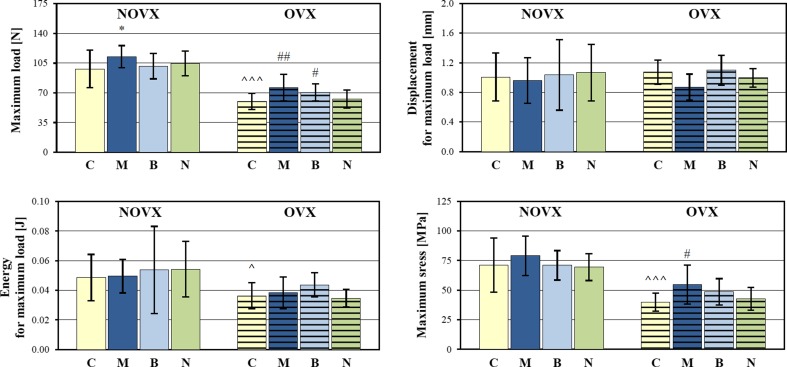
Effects of morphine hydrochloride (*M*; 20 mg/kg *s.c.*), buprenorphine hydrochloride (*B*; 0.05 mg of buprenorphine/kg *s.c.*), and naloxone hydrochloride dihydrate (*N*; 2 mg/kg *s.c.*), administered for 4 weeks, on mechanical properties of the tibial metaphysis (parameters for the maximum load point) in nonovariectomized (NOVX) and ovariectomized (OVX) rats. Results are presented as means ± SD. ^*p* < 0.05, ^^^*p* < 0.001, significant differences between the NOVX and OVX controls (*C*); *p < 0.05, significantly different from the NOVX control rats; ^#^ *p* < 0.05, ^##^ *p* < 0.01, significantly different from the OVX control rats

The mechanical properties of the tibial metaphysis (mostly cancellous bone) were statistically significantly worsened in relation to the NOVX control rats (Fig. 5, Table 3). Both the extrinsic (load, displacement, energy) and intrinsic (Young’s modulus, stress) mechanical parameters were affected. The maximum load and stress as well as load and stress at the fracture point decreased, the corresponding displacement values increased, and the energy absorbed to the maximum load decreased. Similar effects were observed at the yield point (not shown). Young’s modulus was also decreased. Estrogen deficiency did not affect the mechanical properties of cortical bone of the femoral diaphysis. The strength of the femoral neck (built of cortical and cancellous bone) was not significantly affected (Table 3).

**Table 3:** Effects of morphine hydrochloride (20 mg/kg *s.c.*), buprenorphine hydrochloride (0.05 mg of buprenorphine/kg *s.c.*), and naloxone hydrochloride dihydrate (2 mg/kg *s.c.*), administered for 4 weeks, on mechanical properties of the tibial metaphysis (Young’s modulus and the parameters for the fracture point) and on mechanical properties of the femoral diaphysis and femoral neck in nonovariectomized and ovariectomized rats

Parameter/group		Nonovariectomized (NOVX) rats				Ovariectomized (OVX) rats			
		Control	Morphine	Buprenorphine	Naloxone	Control	Morphine	Buprenorphine	Naloxone
Tibia	Young’s modulus (MPa)	1997 ± 606	2026 ± 780	1953 ± 637	1818 ± 507	1209 ± 308^^^	1979 ± 869^##^	1370 ± 495	1402 ± 279
	Fracture load (N)	71.54 ± 17.01	80.64 ± 8.92	78.76 ± 23.62	79.33 ± 17.49	39.07 ± 9.59^^^	46.85 ± 9.31^#^	51.53 ± 8.86^##^	48.58 ± 9.68^#^
	Displacement for fracture load (mm)	1.318 ± 0.365	1.322 ± 0.412	1.408 ± 0.341	1.343 ± 0.381	1.687 ± 0.230^^	1.311 ± 0.247^###^	1.512 ± 0.233	1.390 ± 0.212^#^
	Energy for fracture load (J)	0.075 ± 0.024	0.084 ± 0.019	0.084 ± 0.018	0.079 ± 0.022	0.067 ± 0.009	0.065 ± 0.017	0.069 ± 0.006	0.057 ± 0.012^#^
	Stress for fracture load (MPa)	53.27 ± 24.26	56.89 ± 13.34	55.72 ± 20.11	53.03 ± 14.94	25.97 ± 6.89^^^	33.45 ± 9.84	34.97 ± 6.48^##^	33.19 ± 8.73
Femur	Diaphysis–Young’s modulus (MPa)	11,460 ± 2881	10,922 ± 1949	10,563 ± 1178	10,476 ± 1768	9672 ± 2222	9647 ± 2313	9945 ± 1838	10,257 ± 1304
	Diaphysis–maximum load (N)	101.86 ± 8.69	100.25 ± 11.55	102.50 ± 10.36	101.90 ± 13.70	100.68 ± 12.95	95.53 ± 6.24	101.85 ± 14.15	104.87 ± 10.79
	Neck–maximum load (N)	79.73 ± 5.88	82.62 ± 9.91	78.47 ± 12.63	73.75 ± 20.88	82.72 ± 14.19	83.37 ± 11.38	82.58 ± 7.07	74.70 ± 7.36

Results are presented as means ± SD

^^*p* < 0.01, ^^^*p* < 0.001, significant differences between the NOVX and OVX controls; ^#^ *p* < 0.05, ^##^ *p* < 0.01, ^###^ *p* < 0.001, significantly different from the OVX control rats

### Effects of morphine on the skeletal system

Administration of morphine hydrochloride (20 mg/kg *s.c.*) for 4 weeks to NOVX and OVX rats decreased the body mass gain (Table 1), in relation to the corresponding control rats. There was no effect on the serum concentration of estradiol and the uterus mass/body mass ratio, but after administration of morphine, the thymus mass/body mass ratio significantly decreased. The use of morphine did not affect the serum level of CGRP. Administration of morphine to NOVX and OVX rats reduced the longitudinal growth of the femur (Fig. 3), without affecting bone mass (not shown).

In rats receiving morphine, bone mineralization was intensified, as evidenced by statistically significant increase in the ratio of bone mineral mass to bone mass in the femur of NOVX rats and in the tibia of OVX rats. There was no effect on the content of calcium and phosphorus in the bone mineral (Table 2).

The use of morphine did not statistically significantly affect the serum levels of bone turnover markers (osteocalcin, CTX-I, and TRAP 5b) in NOVX rats (Fig. 4). In OVX rats, concentrations of osteocalcin and CTX-I were statistically decreased. Morphine did not affect the serum calcium level (Table 1).

Administration of morphine to NOVX rats resulted in the increase in the maximum load applied to the tibial metaphysis, without other significant effects on bone mechanical properties (Fig. 5, Table 3). In estrogen-deficient (OVX) rats, Young’s modulus of the tibial metaphysis increased to the values of the NOVX control rats. The maximum load and stress, and the load at the fracture point statistically significantly increased in comparison with the OVX control rats. The values of energy absorbed to these points did not change, compared to the results obtained in the OVX control rats, due to the reduction in the displacement values. There was no effect of morphine on mechanical parameters of the femoral diaphysis and neck (Table 3).

### Effects of buprenorphine on the skeletal system

Administration of buprenorphine (0.05 mg /kg *s.c.*) for 4 weeks to NOVX and OVX rats decreased the body mass gain and did not affect the serum estradiol concentration, and the ratios of mass of the uterus and thymus to the body mass in relation to the corresponding control rats (Table 1). Buprenorphine did not affect the longitudinal growth of the tibia and femur (Fig. 3).

Bone mineralization was improved after administration of buprenorphine (as evidenced by statistically significant increases in the ratio of bone mineral mass to bone mass in the femur of NOVX rats, and in the tibia and L-4 vertebra of OVX rats; Table 2). The content of calcium and phosphorus in the bone mineral was not changed in relation to the appropriate controls (Table 2).

Administration of buprenorphine did not affect the serum bone turnover markers in NOVX rats. In OVX rats, the levels of osteocalcin and CTX-I statistically significantly decreased in comparison to the OVX control rats (Fig. 4). There was no effect of buprenorphine on the serum levels of TRAP 5b and calcium (Table 1).

The use of buprenorphine did not affect the mechanical properties of the tibial metaphysis in NOVX rats (Fig. 5, Table 3). In OVX rats, administration of buprenorphine induced significant increases in the maximum load applied to the tibial metaphysis (Fig. 5) and in the load and stress at the fracture point (Table 3), counteracting the effects of estrogen deficiency. Administration of buprenorphine did not affect the mechanical properties of the femoral diaphysis and neck both in NOVX and OVX rats (Table 3).

### Effects of naloxone on the skeletal system

Administration of naloxone hydrochloride dihydrate (2 mg/kg *s.c.*) for 4 weeks to NOVX and OVX rats did not affect the body mass gain, the serum concentration of estradiol, and the ratios of the uterus and thymus mass to the body mass in relation to the corresponding control rats (Table 1). Naloxone did not affect the longitudinal growth of the tibia and femur (Fig. 3).

Administration of naloxone in NOVX rats induced increases in the ratio of bone mineral mass to bone mass in the femur (statistically significant) and L-4 vertebra (Table 2). There was no effect of naloxone on the bone mineral mass/bone mass ratio in OVX rats. Naloxone did not significantly affect calcium and phosphorus content in the bone mineral (Table 2).

Administration of naloxone did not affect the serum bone turnover markers and calcium levels both in NOVX and OVX rats (Fig. 4, Table 1).

Naloxone did not affect the mechanical properties of the tibial metaphysis in NOVX rats (Fig. 5, Table 3). In OVX rats, the fracture point load increased and the values of displacement and energy decreased in relation to the OVX control rats (Table 3). Administration of naloxone did not affect the mechanical properties of the femoral diaphysis and neck both in NOVX and OVX rats (Table 3).

## Discussion

The effects of drugs acting through opioid receptors on the skeletal system, investigated in the present study, did not confirm the damaging action of opioid analgesics, which might have been expected based on the literature. In fact, beneficial effects of morphine and buprenorphine in rats with estrogen deficiency were observed. A similar, though weaker, effects were present in rats with normal estrogen levels. Administration of an opioid receptor antagonist, naloxone, did not exert opposite effects to those induced by the investigated opioid analgesics.

The only unfavorable effect of morphine on the skeletal system in the present study was the inhibition of the longitudinal growth of the tibia and femur, concurrently with the decrease in body mass gain. The inhibition was observed both in NOVX and OVX rats. Consistently, a reduction in the number of cells in the proliferation zone of the growth plate (and the growth plate width) under the influence of morphine was demonstrated in young rats (Ezzatabadipour et al. 2011). Also, a restriction of fetal growth often occurs during pregnancy in opioid-dependent women, although numerous studies have demonstrated the stimulatory effect of opioids on the secretion of growth hormone (Olsen et al. 2014). Neither buprenorphine, a partial μ receptor agonist and κ receptor antagonist, nor naloxone, an opioid receptor antagonist, affected the longitudinal bone growth. Also, bone growth was not affected in Dyn−/− mice lacking dynorphin (which act mainly through κ receptors) expression (Baldock et al. 2012).

Morphine and buprenorphine counteracted the development of osteoporotic changes in the skeletal system of OVX rats. Estrogen deficiency in rats, similarly as in postmenopausal women, leads to increased bone remodeling processes with the predominance of resorption over formation, and cancellous bone is more affected than compact bone (Lelovas et al. 2008). This was confirmed in the present study; both bone formation and bone resorption were increased, and bone mineralization was impaired in OVX rats. These changes led to worsening of mechanical properties of cancellous bone of the tibial metaphysis.

After administration of the opioid agonists to OVX rats, bone turnover rate slowed down, as demonstrated by significant reduction of bone formation and bone resorption markers. Also, bone mineralization was improved. These activities led to the increase in the strength of the tibial metaphysis (cancellous bone). Both drugs did not affect mechanical properties of the femoral diaphysis (cortical bone) and neck (cortical and cancellous bone). The effects of opioid agonists on the skeletal system of NOVX rats were limited to the improvement of bone mineralization (morphine and buprenorphine) and mechanical properties of cancellous bone (morphine).

Although long-term use of opioids in humans leads to the development of hypogonadism, administration of morphine, buprenorphine, and naloxone in the present study did not affect the serum estradiol level and uterus mass/body mass ratio. In fact, the skeletal effects of morphine and buprenorphine in OVX rats were similar to those induced by estradiol supplementation in our previous studies (Folwarczna et al. 2009; Cegieła et al. 2012). Taken together, drugs acting through opioid receptors did not significantly affect the systemic estrogen levels in rats.

Results of the present study are at variance with the widely held view that opioid analgesics unfavorably affect the skeletal system in humans, based on most of previous reports. There are epidemiological data indicating the decreased BMD in patients treated with opioids in relation to nontreated controls (Kim et al. 2006; Dürsteler-MacFarland et al. 2011; Grey et al. 2011; Duarte et al. 2013). Interestingly, the decreased BMD during methadone maintenance therapy was observed in male patients only (Grey et al. 2011). However, those studies did not determine the changes in the skeletal system, and the data were rather not adjusted for other factors, like the treated diseases and poor general health status. Other studies demonstrated that the use of opioid analgesics is associated with an increased risk of fractures, to which the increased risk of falls may contribute (Ensrud et al. 2003; Vestergaard et al. 2006; Saunders et al. 2010; Miller et al. 2011; Carbone et al. 2013; Li et al. 2013).

Nevertheless, results of the present study are consistent with recently published reports on the possible favorable opioid effects on the skeletal system in women (Vestergaard et al. 2012; Lee et al. 2013). A small trend to a smaller decline in the spine bone mineral density over 10 years in postmenopausal women using opioids, in comparison to non-exposed, was demonstrated (however with a nonsignificant trend towards more fractures) in Danish Osteoporosis Prevention Study (Vestergaard et al. 2012). It was also demonstrated in a population-based nested case-control study that morphine tends to exert the protective action on the skeletal system of female patients with cancer. The use of morphine in patients treated with bisphosphonates significantly reduced the risk of osteoporosis (compared with patients on bisphosphonates only) (Lee et al. 2013). Results of these studies indicate on the possibility of favorable direct opioid effects on bones.

Previous experimental studies demonstrated rather bone-damaging effects of opioid receptor agonists. The development of osteoporosis in rats was reported after 3-month administration of morphine at a lower dose (Boshra 2011); the author associated the skeletal changes with reduced levels of estrogen, not observed in the present study. Morphine unfavorably affected the skeletal system in mice with experimental sarcoma, with no influence on control animals (King et al. 2007). The inhibition of fracture healing by morphine was demonstrated in rats; this effect, however, was associated with inhibition of callus resorption (Chrastil et al. 2013). Morphine inhibited the orthodontic movement of teeth in rats, which also indicates the inhibition of bone resorption (Akhoundi et al. 2010), consistently with results of the present study (inhibition of bone resorption by morphine and buprenorphine).

The mechanism of the beneficial effect of morphine and buprenorphine observed in the present study may be associated with their direct effects on opioid receptors in bone cells. All opioid receptors were demonstrated to be present in human osteoblast-like MG-63 cells (Pérez-Castrillón et al. 2000). The stimulation of opioid receptors leads to the inhibition of adenylate cyclase and reduction of the cellular cAMP level (Al-Hasani and Bruchas 2011). Since increased cAMP levels (for example induced by parathormone) activate cAMP-dependent protein kinase A in marrow stromal cells and osteoblasts, inducing both bone formation and bone resorption (the latter by stimulating the secretion of RANKL) (Kondo et al. 2002; Wang et al. 2006), it is possible that decreased bone turnover induced by the opioid analgesics may be due to the reduced level of cAMP in osteoblasts. Similar actions of morphine (an agonist of μ, δ, and κ receptors) and buprenorphine (a partial μ receptor agonist and κ receptor antagonist) suggest the responsibility of μ receptors for the observed effects. Also, based on the results of Baldock et al. (2012), the antagonistic effect on κ receptors could contribute to the beneficial effects of buprenorphine in present study.

In search of the mechanism of action of the opioid analgesics on bones, serum CGRP levels were determined. CGRP, apart of the involvement in the pain transmission and modulation, directly affects bone cells, increasing bone formation and decreasing bone resorption (Mrak et al. 2010). Although serum CGRP levels in OVX rats slightly increased in relation to NOVX controls, in accordance with higher CGRP levels in postmenopausal than in premenopausal women (Gupta et al. 2008), there was no effect of the opioids on the serum CGRP level.

A surprising result of the present study is the observation that naloxone not only did not exert opposite to morphine and buprenorphine effects but also affected some parameters similarly to the opioid receptor agonists. The administration of naloxone to OVX rats increased the fracture point load in cancellous bone of the tibial metaphysis; in NOVX rats, there was an increase in the femur mineralization. The improvement of some bone parameters induced by naloxone in rats is consistent with the effect of administration of low-dose naloxone in the sheep: increased mineralization and callus remodeling in a model of bone damage (Petrizzi et al. 2007). However, the reports on effects of opioid receptor antagonists on bone resorption are inconsistent; naltrexone caused a decrease of orthodontic tooth movement in rats with experimentally induced cholestasis (Nilforoushan et al. 2002), and the topical application of naloxone intensified alveolar bone loss in experimentally induced periodontitis in rats (Queiroz-Junior et al. 2013).

The lack of opposing effect of naloxone in relation to morphine and buprenorphine may be due to the fact that the naloxone dose chosen as the dose blocking opioid receptors, might lead to agonistic effects within the skeletal system. It has been shown that naloxone in small doses has an analgesic effect in humans and animals (Petrizzi et al. 2007). Due to the enormous complexity of the endogenous opioid peptide system, and opioid receptors, it is possible that the use of naloxone at a particular dosage may differentially modify the action of individual peptides. For example, the Dyn−/− mice had increased bone formation markers, which indicates that dynorphin inhibits bone formation (Baldock et al. 2012). Thus, blocking of κ receptors by naloxone and buprenorphine may lead to improvement of certain parameters related to bone formation.

The significance of the study is that it for the first time reports on the favorable effects of opioid receptor agonists (morphine and buprenorphine), used in the therapeutic dose range, on the skeletal system in female rats, especially in conditions of estrogen deficiency. Results of the study, indicating the improvement in bone mechanical properties and mineralization by the opioid analgesics, support and provide the experimental background for the isolated so far observations made in female patients indicating some positive skeletal effects of opioids on bone (Vestergaard et al. 2012; Lee et al. 2013). Based on the results of the present study, it seems likely that opioids also in humans may not only not damage the skeletal system but also exert some direct favorable effects, which are counteracted by indirect detrimental ones. This seems to be important in the light of numerous, both reasonable and unfounded, concerns over the use of the opioid analgesics in the long-term therapy of pain.

The limitation of the study is that it concerns the experimental conditions in rats only and does not provide the data on the bone microstructure. Moreover, since the three drugs differently affecting opioid receptors were investigated at one dose each, and there was one duration (4 weeks) of drug administration, results of the study do not exclude the possibility of unfavorable skeletal effects of opioids used in larger doses and for longer duration. It is also possible that the beneficial effects of opioid analgesic drugs may be limited to females. Taken together, the study may be treated as preliminary one.

In conclusion, results of the present study confirmed the role of opioid receptors in the regulation of bone remodeling processes and demonstrated, in experimental conditions, that the use of opioid analgesics at moderate doses may have beneficial effects on the skeletal system, especially in estrogen deficiency. Since the favorable opioid effects were demonstrated in female rats, and some positive skeletal effects were reported in female patients, it seems interesting to answer the question whether the effects of opioid analgesics on the skeletal system depend on sex.
